# Application of a Quick Response Code as an Alternative Method to Provide Pediatric Cast Care Instructions

**DOI:** 10.5435/JAAOSGlobal-D-20-00105

**Published:** 2020-07-02

**Authors:** Michael Basso-Williams, Katie Fletcher, Bryn R. Gornick, Kevin Kwan, John A. Schlechter

**Affiliations:** From the Department of Orthopedics, Riverside University Health Systems, Moreno Valley, CA (Dr. Basso-Williams and Dr. Schlechter); the Department of Orthopedics, Children's Hospital Orange County (Ms. Fletcher, Ms. Gornick, and Dr. Schlechter), the Pediatric Orthopedic Specialists of Orange County (Ms. Fletcher,Ms. Gornick, and Dr. Schlechter), Orange, CA; and the College of Osteopathic Medicine of the Pacific, Western University of Health Sciences, Pomona, CA (Mr. Kwan).

## Abstract

**Background:**

There is a growing need to improve patient education for nonsurgical fracture care in children. A Quick Response (QR) code was used as an alternative method to provide cast care instructions in our outpatient fracture clinic. We evaluated satisfaction and examined the convenience and impact this might have on the child's casting experience.

**Methods:**

A prospective study was conducted in which QR codes were embedded in the casting of nonsurgical pediatric fractures in 88 children. The number of times the QR code was scanned, who scanned the code, treatment satisfaction, cast-related issues, and whether scan helped prevent a call to the treating physician were recorded.

**Results:**

Google Analytics showed the QR code was scanned an average of 1.6 times by 60 participants with most scans done by a parent (65%). Seventy-nine participants (89.9%) found it useful to have the QR code on their cast, and 65 (73.9%) were “very satisfied” with the convenience of the QR code and 37 stated that the information they found kept them from contacting the physician.

**Discussion:**

We demonstrated that the use of QR codes for nonsurgical pediatric fracture care has a high level of satisfaction and may reduce calls to the treating physician.

Because of the dynamic nature of the American healthcare system, an inherent need for efficient and cost-effective delivery of medical information exists while still providing exceptional patient care. DiPaola et al,^1^ reported that unplanned cast changes in the pediatric population are “because of patient nonadherence to instructions and not the result of cast application technique.” To overcome this obstacle, we investigated new ways to provide more accessible and technologically advanced physician-recommended guidelines and instructions to promote compliance and ultimately reduce unwarranted complications during pediatric casting regimens.

To accomplish this goal, a new technological approach for the advancement of clinical practice in orthopaedic settings using a Quick Response (QR) code was used. A QR code can provide patients with real-time access to educational information regarding their medical care.^2-4^ DiPaola et al^1^ suggested that their educational efforts when using verbal and written instructions to prevent cast complications in the pediatric population were not adequate. Scanning a QR code on a cast, splint, brace, or other similar device allows the patient to have continuous access to guidelines regarding their care that can promote patient compliance and improve the progression of one's treatment.^2^

The purpose of our study was to evaluate the children and families' satisfaction and examine the utility and usefulness a QR code might have on the child's experience while in cast.

## Methods

**Figure 1:**
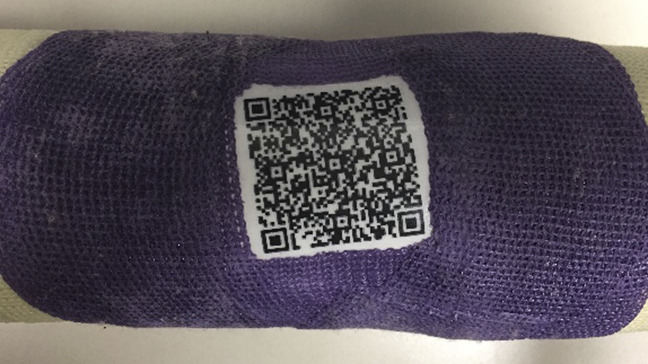
Photograph representing a Quick Response code as displayed on a pediatric cast.

Children between the ages of 0 to 18 years with an upper or lower extremity nonsurgical fracture were chosen to participate in this Institutional Review Board (IRB)-approved study. Children were excluded if they had a previous history of casting. A member of their family—patients, parents, or their legal guardian—had to own a smartphone to be included. On meeting the criteria, agreement and signed consent, a laminated QR code was placed directly onto the patient's cast. The peripheral borders of a laminated, waterproof QR code measuring 37 mm × 37 mm was secured into the cast with fiberglass cast material (Scotchcast Plus Casting Tape; 3M) (Figure 1).

The QR codes placed met the family's primary language, English or Spanish. The child or their parents were instructed to download a free QR code scanning application to their smartphone. Scanning of the QR code would link directly to a website displaying cast care instructions in either English or Spanish. Here, they would have the option to download the cast care instructions or read through the provided cast care information. The URL linked to the QR code was https://www.choc.org/orthopaedics/cast-care-instructions/?utm_source=cast&utm_medium=qrcode&utm_campaign=cast-care&utm_term=cast-care-english. In addition to instructions on how to operate the QR code, patients and families were also given verbal cast care instructions. No written instructions were provided at the time of cast placement.

On returning to the outpatient orthopaedic clinic for their follow-up appointment, they were evaluated for any cast-related issues or the QR code scanning ability. Cast-related issues included wet casts, a broken cast, a skin irritation, and early cast removal. They were given a questionnaire at the time of cast removal to document and state the number of times they referred to the given information, if it was found useful to have a QR code, and any additional feedback they were willing to provide. The questionnaire was written so that no more than 5 years of education would be needed to completely understand the passage/questions. Although families were not asked to specify why they scanned the code, the questionnaire did ask “Did you ever scan the code when you had a question about how to take care of your child's cast?” If they answered yes to this question, there was a follow-up question “If yes, did scanning the code prevent a call to the medical team?” This differentiated patients who scanned the code to see how QR codes worked and patients who needed to scan the code to receive medical advice and determined whether there was a reduction in calls to the treating physician.

Google Analytics was used to track the number of scans from each unique QR code embedded. Each code was specific to the patient, allowing us to track the number of times each code was scanned, not the number of devices used to scan the codes. Analysis of Google Analytics reports was used to investigate the utility of the QR code application. QR code placement and follow-up surveys were collected between December 2017 and April 2019.

The distressed communities index (DCI), a single measure of economic well-being that encompasses seven metrics to provide a clear depiction of the economic and social state of various zip codes, counties, and cities, was examined. Metrics that help determine DCI include education level, unemployment, median income, poverty rate, housing vacancies, and business growth. In addition, the distress tier, a measure of economic well-being per city, was collected based on the patient's zip code.^5^ An analysis of the distress tier and DCI of the child and family was done to evaluate any relationship between the level of the distress tier and DCI and number of cast-related issues. Smartphone type and the incidence of cast-related issues were also evaluated.

Descriptive statistics were calculated for all variables. Paired samples Student *t*-test was used to compare the stated number of times a code was scanned versus the actual number of times a code was scanned. Chi square analysis was used to compare categorical outcomes between patients who scanned the code versus those who did not scan. Analysis of variance (ANOVA) was used to compare averages for continuous outcomes based on categorical independent variables. Continuous variables were checked for normality and homogeneity of variances before utilization of parametric statistics. Spearman rho correlation was used to evaluate for a relationship between the distressed community index and the actual number of times the QR code was scanned. Analyses were performed using SPSS v. 25, and alpha was set at *P* < 0.05 to declare significance (IBM Copr. Released 2017. IBM SPSS Statistics for Windows, Version 25.0; IBM Corp.).

## Results

A total of 100 participants were enrolled in the study, and 88 participants (children or family member) completed the follow-up questionnaire and were used for analysis (Table 1). Patients who did not complete the follow-up survey before May 2019 were not included in the study. The mean age was 8.2 years with an age range between 9 months and 17 years of age. Male patients comprised 59.1% of the population (n = 52) and the remaining 40.9% (n = 36) were female patients. Most casts placed were short arm casts (40.9%), followed by long arm casts (33%) and short leg casts (21.6%). The remaining cast types were long leg (4.5%). The type of injury sustained has been outlined in Table 2. Only two casts with a QR code were placed by a physician, and the remaining casts were placed by an orthopaedic technician.

**Table 1:** Study Values and Statistical Significance

Demographics and Study Findings	Values	*P* Value
Participants	88	—
Age range (yr)	0-17	—
Average age (yr)	8.19	—
Average number of times code was scanned	1.56	<0.001
Perceived number of times the code was scanned	3.74	
Percent of participants that scanned the code	68.2%	
Found the code convenient and useful	89.7%	<0.001
Very satisfied with the convenience	73.9%	0.02
Children with a cast-related issue	12.5%	0.299
Participants who had difficulty with the QR code	9.1%	

QR = Quick Response

**Table 2:** Fracture Location and Frequency

Fracture Location	Values
5th metatarsal	3
Distal fibula	12
Distal tibia	1
Shaft of tibia	5
Femur	1
Distal humerus	1
Supracondylar humerus	19
Lateral condyle	1
Medial epicondyle	1
Olecranon	2
Radial shaft	2
Ulnar shaft	1
Distal radius	35
Distal radius and ulna	2
First metacarpal	1
Proximal phalanx	1
Total	88

Of the participants (children or family member), 72.7% reported their primary language as English and were given a QR code taking them to the English instructions. The remaining 27.3% reported their primary language as Spanish and were given a QR code taking them to the Spanish instructions. Of the 88 participants, 60 patients scanned the code. The QR code was scanned an average number of 1.6 times (range, 0 to 8), although the perceived number of scans was 2.4 times higher (range, 0 to 15). This was found to be statistically significant with a *P* < 0.001. Most of the scans (64.8%) were made by the parents and the remainder were made by the child, a sibling, or another unspecified relationship to the patient. Thirty-seven of the 60 participants who scanned the code (42% of the total cohort) reported that the information they found prevented them from needing to call the medical team (*P* < 0.0001).

Eleven cast-related issues were reported. Seven participants reported an episode of a wet cast on the questionnaire they were provided at their scheduled clinic visit, there was one reported occurrence of rash/skin maceration without a wet cast, one cast was removed by the child's father the day before the scheduled cast removal secondary to the cast getting wet, one damaged cast, and one cast that fell off prematurely. Of the 11 cast-related issues, 55% of the patients or families scanned the QR code. However, it was not reported whether they scanned the code before or after their cast-related issue took place. Of the patients who never had their QR code scanned (28), five sustained a cast-related issue (18%). Twenty-six percent of the cohort never had their QR code scanned and did not sustain a cast-related issue, whereas 62% had their code scanned and did not sustain a cast-related issue.

Two of the eight reported wet casts led to an unexpected cast change (2.3%). Both of these unexpected cast changes occurred because of short leg casts becoming wet. Those who did not have their casts changed secondary to reporting a wet cast simply had their cast removed (n = 5) and reassurance was provided or had their cast removed and were transitioned to a functional brace (n = 3). Fifty-four percent (n = 6) of the 11 cast-related issues were associated with lower extremity casts (long and short leg).

Seven participants reported a problem with the QR code. Three reported the code would not scan, two reported the code fell off the cast, one reported they were unable to find the code on the cast, and one reported the code stopped working past the first scan.

**Figure 2:**
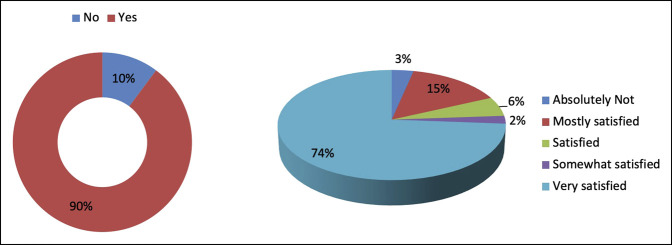
**A**, Pie Chart representing patients who found the Quick Response Code convenient and easy to use. **B**, Patient satisfaction.

Despite these issues, 89.7% (n = 79) of the participants found it convenient and useful to have the QR code on their cast (*P* < 0.001) and 73.9% (n = 65) of the patients were “very satisfied” with the convenience associated with having a QR code on their cast (*P* = 0.02), as seen in Figure 2.

An analysis on the patient's distress tier and DCI leading to an increased incidence in cast-related issues was not found to be statistically notable in this cohort. Furthermore, no statistical significance was noted concerning patients who scan the code as often or not at all having higher complications than those who scan the code more often (*P* = 0.364). Descriptive statistics were run to determine whether the type of smart phone used to scan the code was associated with complication rates. A *P*-value of 0.574 indicated that no correlation was found between either smartphone type or the incidence of cast-related issues.

## Discussion

The application of QR codes on pediatric patients presenting with nonsurgical fractures requiring casting was tested in a clinical setting and has the potential to expand across subspecialties. This investigation presents a novel approach for the advancement of clinical practice and serves as a foundation for future studies involving QR codes in clinical and research applications. This approach promotes personalized medicine by providing the participants (children or family member), with real-time access to information regarding their treatment and appropriate cast care. As a result, this study establishes some benchmark values for the use of QR technology for children presenting with nonsurgical fractures where a cast was recommended by a physician as the primary treatment method.

To our knowledge, no study has been done focusing solely on the pediatric population and the use of a QR code to provide easily accessible, cost-effective cast care instructions. Gough et al^3^ published a study where a QR code was implemented with telephone numbers for use in providing cast care instructions. However, this study focused on an older population age (mean age 50 years) with only 56% of the cohort owing a cell phone. Of the 56% that owned a cellular phone, 33% of those patients scanned the code.^3^ Our study supports that QR codes for proper cast maintenance can also be effective in mitigating potential cast complications in a pediatric population as well. Despite younger age being a risk factor for cast complications, the use of QR codes can reduce cast-related issues while also maintaining patient satisfaction.^6^

Not only does the proposed treatment method allow children and family members to receive personalized care at their fingertips, it takes advantage of the growing technology era.^3,4^ Currently, 96% of Americans own a cellular phone of some kind, whereas 81% own a smartphone to keep up with their “on the go” lifestyle.^7^ Findings from Smith, *U.S. Smartphone Use in 2015*, reported that 62% of smartphone users have used their phone in the past year to look up information about a health condition.^8^

A number of studies have suggested that only 50% of information presented to patients during their office visit is retained.^9^ More alarmingly, 40% to 80% of information provided to the patient by healthcare practitioners is forgotten immediately.^9^ According to Kessels, *Patients' memory for medical information*, “patients tend to focus on diagnosis-related information and fail to register instructions on treatment.”^10^ Owing to the child's and families' anxiety and distress concerning the broken bone, it is often difficult for the child and family to recall important cast care recommendations with some remembering as little as 14% of spoken instructions.^3,10^ To overcome this challenge, a QR code implemented directly onto a cast allows the child and family to access valuable information at their convenience when questions arise.

Of the 88 casts with QR codes embedded, 60 patient's codes (68.2%) were scanned to gain more information about appropriate cast care when questions arose about how to take care of the child's cast. From these 60 participants, 37 stated that the information they found on the website kept them from needing to contact the treating physician to ask a cast care question that resulted in a reduction of calls to the medical team and was found to be statistically notable.

A total of 11 participants reported a cast-related issue, with most (n = 8) concerning wet casts. In a study done by Sawyer et al,^11^ they noted that of the 168 cast-related emergency department visits that occurred, 29% of them were because of wet casts.^1^ However, in our study, wet casts as a cast-related issue was 9%. Two of the eight reported wet casts led to unexpected cast changes (2.3%) in our study versus 5.3% in a pediatric population as observed in a previous study by Dipaola et al.^1^ Given the frequency of wet casts as a complication, including specific simple cost-effective methods of preventing wet casts as part of the QR code information may be beneficial.^2,12^ As there was a decrease in wet cast–related issues and unexpected cast changes compared with the study fo Dipaola, the use of a QR code may be beneficial for providing pediatric cast care instructions compared with written instructions.

No statistical correlation to the cast complication rate and the number of times the QR code was scanned was present, if it was scanned at all. Of the 11 patients with a cast complication, seven still reported that they were very satisfied with the QR code form of treatment and only one reported they were “absolutely not” satisfied. The remaining three were either “mostly satisfied” (n = 2) or “satisfied” (n = 1).

The results of this study showed that 73.9% (n = 65) of the participants were “very satisfied” with this treatment method and only 3.4% (n = 3) were “absolutely not” satisfied with this treatment method. The remainder of participants were mostly satisfied, satisfied, or somewhat satisfied. These results further support the findings in the study of Gough et al, suggesting that 84% of patients were reassured by the presence of a QR code with telephone numbers on their cast.^2^

Although cast care instructions are readily available on the internet, it can often be challenging to navigate which recommendations are accurate and applicable to each child's specific situation or concern. Implementing a QR code allows the child and family to access physician-specific information that provides reassurance and ensures they are receiving reliable information. This same methodology can be applied to a variety of medical disciplines requiring compliance to specific physician-recommended instructions.^2-4^

Our data demonstrate that the use of QR codes for nonsurgical pediatric fracture care have a high level of satisfaction, the ability to reduce calls to the treating physician as reported by patients and their families, and are noninferior to written and/or verbal instructions. This study provides a solid foundation and encouraging results supporting the need for a larger cohort, multicenter study in the pediatric population to determine the effectiveness of QR code use and the potential reduction in cast care–related issues.
